# Supplementary material for: Modeling the Interactions Between Chemicals and Proteins to Predict the Health Consequences of Air Pollution
Source: Int J Environ Res Public Health. 2025 Mar 13;22(3):418. doi: 10.3390/ijerph22030418 (PMC11942511; doi:10.3390/ijerph22030418)
Supplement: Supplementary file 1 [file ijerph-22-00418-s001.zip › ijerph-3441231-supplementary.pdf]

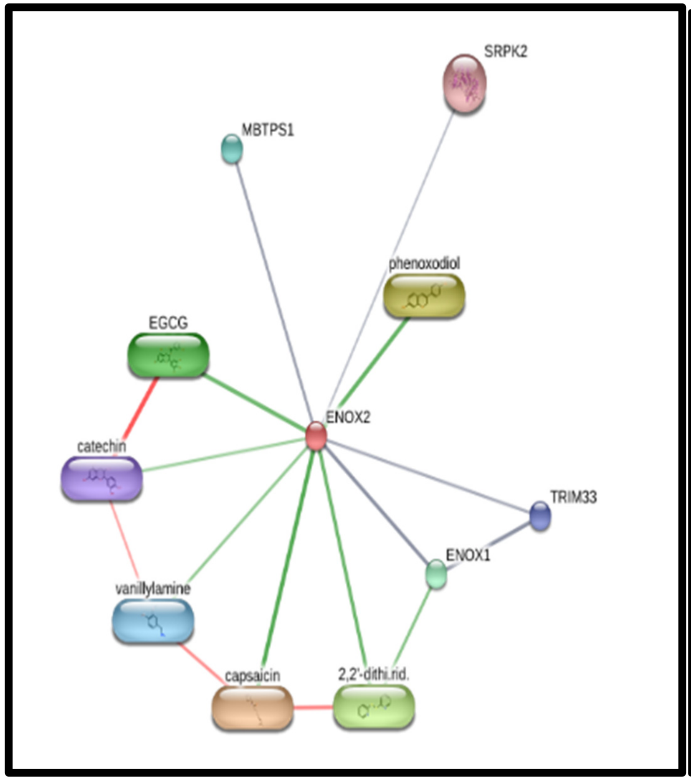
SRPK2
MBTPS1
phenoxodiol
EGCG
ENOX2
catechin
TRIM33
ENOX1
vanillylamine
capsaicin
2,2'-dithi.rid.

(NOX)

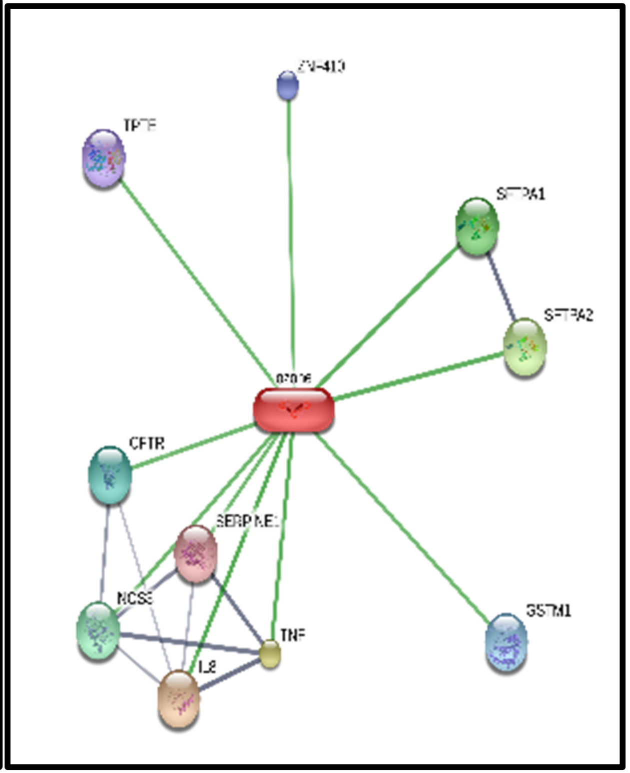
SFTPA1
SFTPA2
CFTR
TNF
GSTM1

(O3)

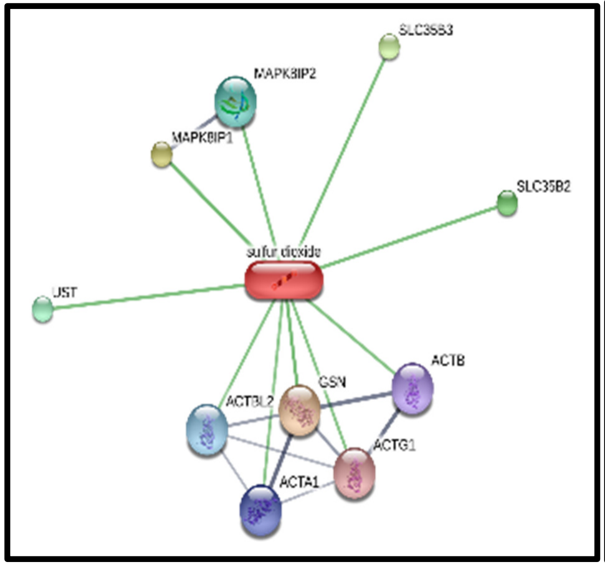

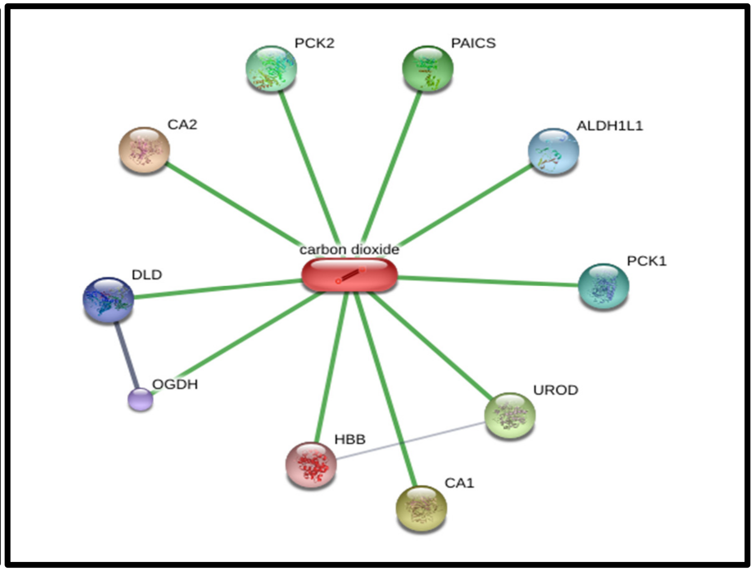

Volatile CO_2 SO_2

Figure S1. Nitrogen oxides (NO_x), Ozone (O_3), Volatile CO_2, and Sulfur dioxide (SO_2) with human proteins were found using STITCH 4.0.

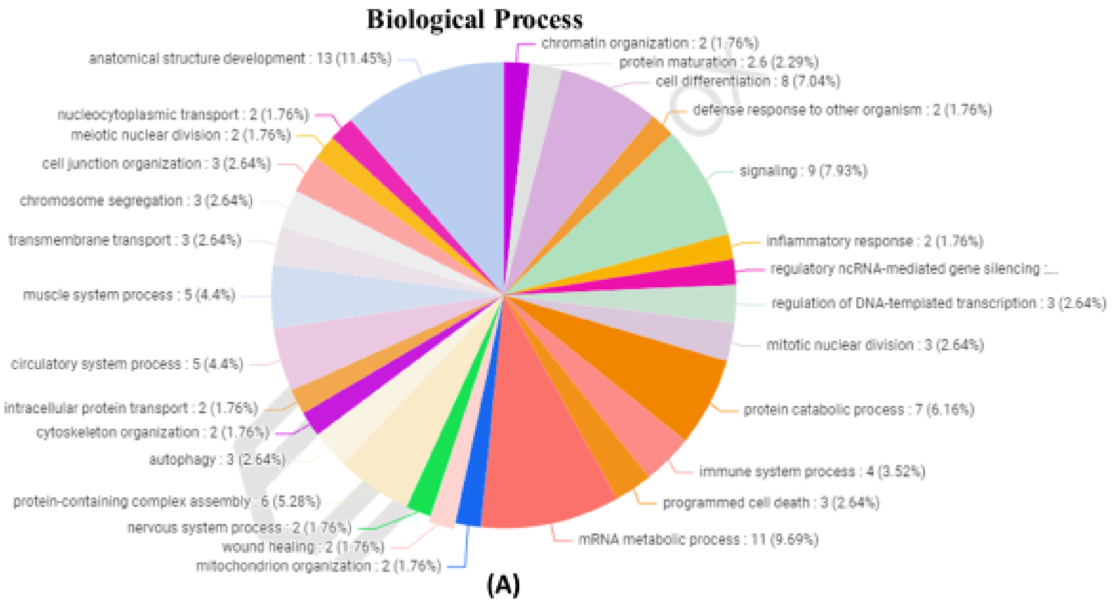
Biological Process
anatomical structure development : 13 (11.45%)
chromatin organization : 2 (1.76%)
protein maturation : 2.6 (2.29%)
cell differentiation : 8 (7.04%)
defense response to other organism : 2 (1.76%)
nucleocytoplasmic transport : 2 (1.76%)
meiotic nuclear division : 2 (1.76%)
cell junction organization : 3 (2.64%)
chromosome segregation : 3 (2.64%)
signaling : 9 (7.93%)
transmembrane transport : 3 (2.64%)
inflammatory response : 2 (1.76%)
regulatory ncRNA-mediated gene silencing :...
muscle system process : 5 (4.4%)
regulation of DNA-templated transcription : 3 (2.64%)
mitotic nuclear division : 3 (2.64%)
circulatory system process : 5 (4.4%)
intracellular protein transport : 2 (1.76%)
protein catabolic process : 7 (6.16%)
cytoskeleton organization : 2 (1.76%)
autophagy : 3 (2.64%)
immune system process : 4 (3.52%)
protein-containing complex assembly : 6 (5.28%)
programmed cell death : 3 (2.64%)
nervous system process : 2 (1.76%)
mRNA metabolic process : 11 (9.69%)
wound healing : 2 (1.76%)
mitochondrion organization : 2 (1.76%)

(A)

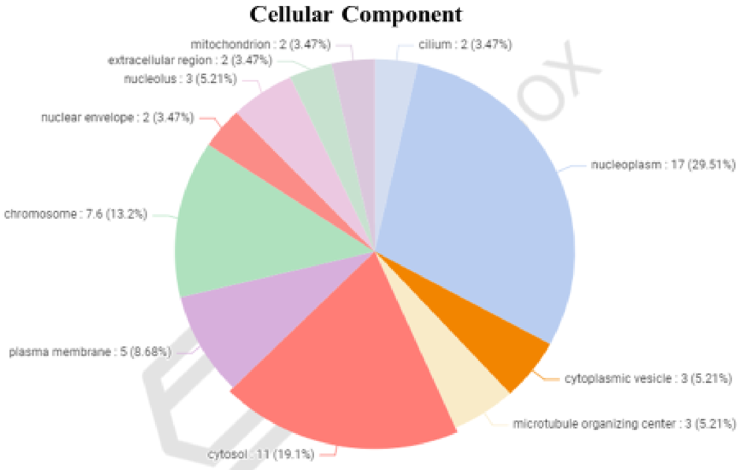
Cellular Component
mitochondrion : 2 (3.47%)
cilium : 2 (3.47%)
extracellular region : 2 (3.47%)
nucleolus : 3 (5.21%)
nuclear envelope : 2 (3.47%)
nucleoplasm : 17 (29.51%)
chromosome : 7.6 (13.2%)
plasma membrane : 5 (8.68%)
cytoplasmic vesicle : 3 (5.21%)
microtubule organizing center : 3 (5.21%)
cytosol : 11 (19.1%)

(B)

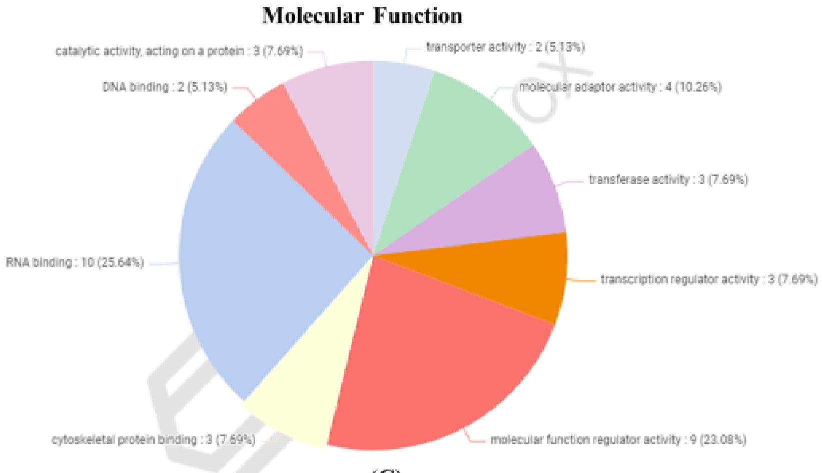

(C)

Figure S2. Functional annotation study revealed NO*x*'s impacts on diverse biological processes (a), cellular component localization (b), and molecular functions (c) of distinct proteins in *Homo sapiens*.

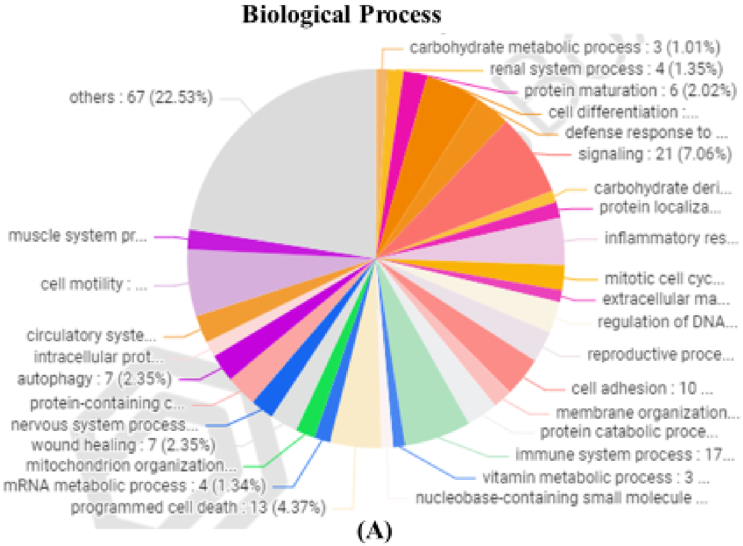

(A)

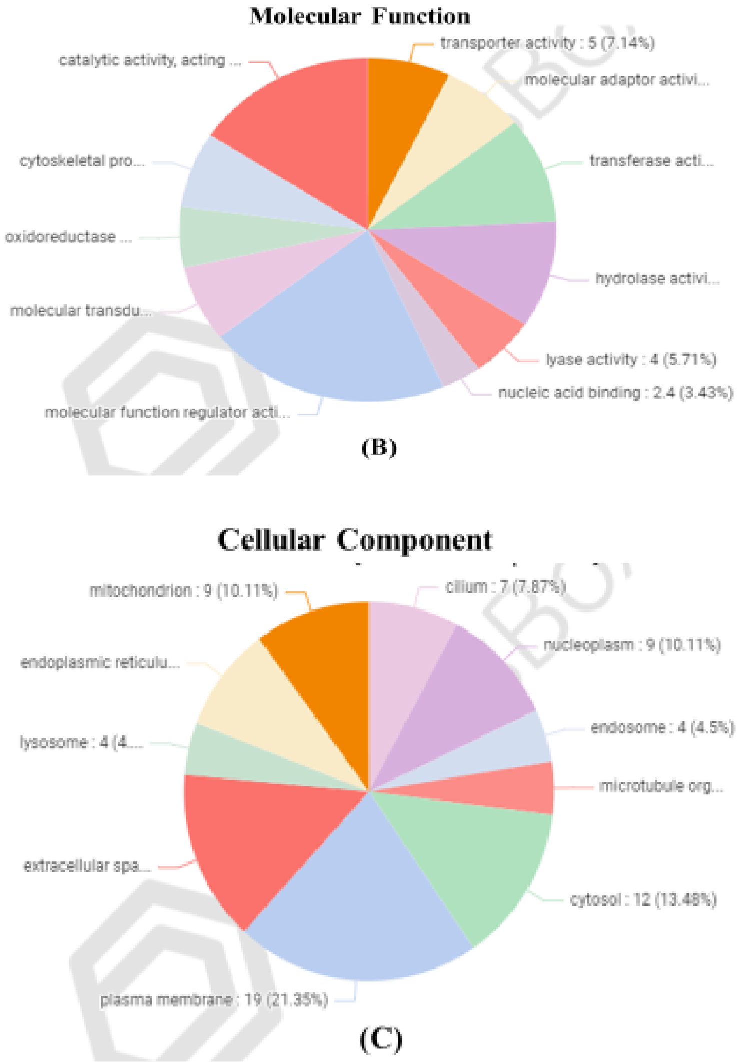

Figure S3. Functional annotation study revealed Ozone (O_3)'s impacts on diverse biological processes (A), molecular functions localization (B), and Cellular Component (C) of distinct proteins in *Homo sapiens*.

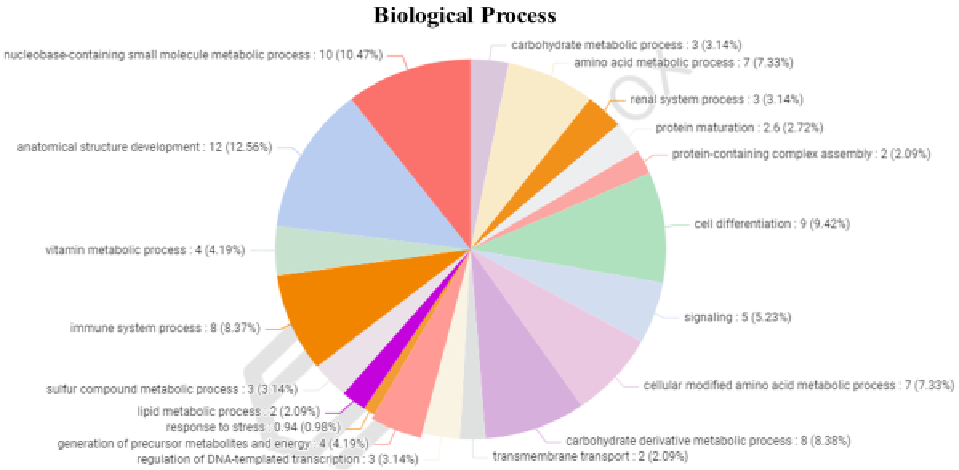
Biological Process
nucleobase-containing small molecule metabolic process : 10 (10.47%)
carbohydrate metabolic process : 3 (3.14%)
amino acid metabolic process : 7 (7.33%)
renal system process : 3 (3.14%)
protein maturation : 2.6 (2.72%)
protein-containing complex assembly : 2 (2.09%)
anatomical structure development : 12 (12.56%)
cell differentiation : 9 (9.42%)
vitamin metabolic process : 4 (4.19%)
signaling : 5 (5.23%)
immune system process : 8 (8.37%)
cellular modified amino acid metabolic process : 7 (7.33%)
sulfur compound metabolic process : 3 (3.14%)
lipid metabolic process : 2 (2.09%)
response to stress : 0.94 (0.98%)
generation of precursor metabolites and energy : 4 (4.19%)
regulation of DNA-templated transcription : 3 (3.14%)
carbohydrate derivative metabolic process : 8 (8.38%)
transmembrane transport : 2 (2.09%)

(A)

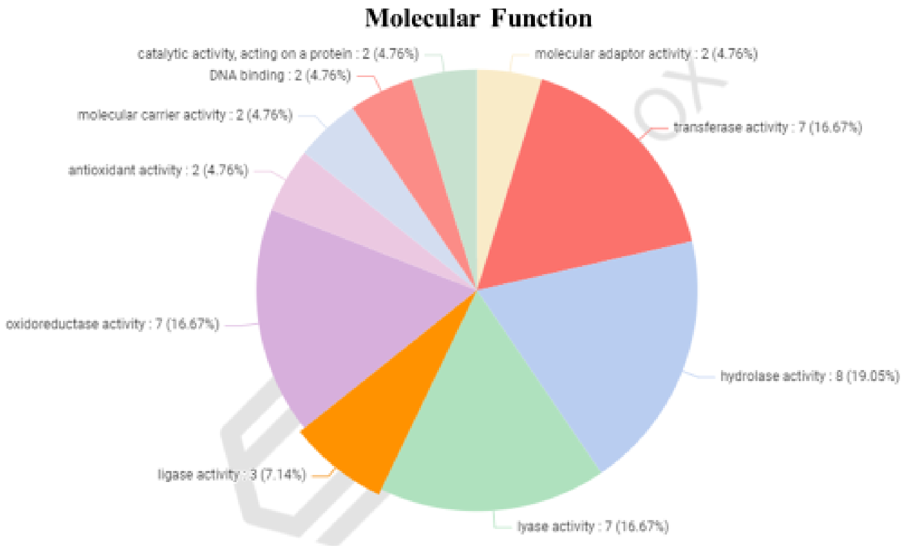
Molecular Function
catalytic activity, acting on a protein : 2 (4.76%)
DNA binding : 2 (4.76%)
molecular carrier activity : 2 (4.76%)
antioxidant activity : 2 (4.76%)
oxidoreductase activity : 7 (16.67%)
ligase activity : 3 (7.14%)
lyase activity : 7 (16.67%)
hydrolase activity : 8 (19.05%)
transferase activity : 7 (16.67%)
molecular adaptor activity : 2 (4.76%)

(B)

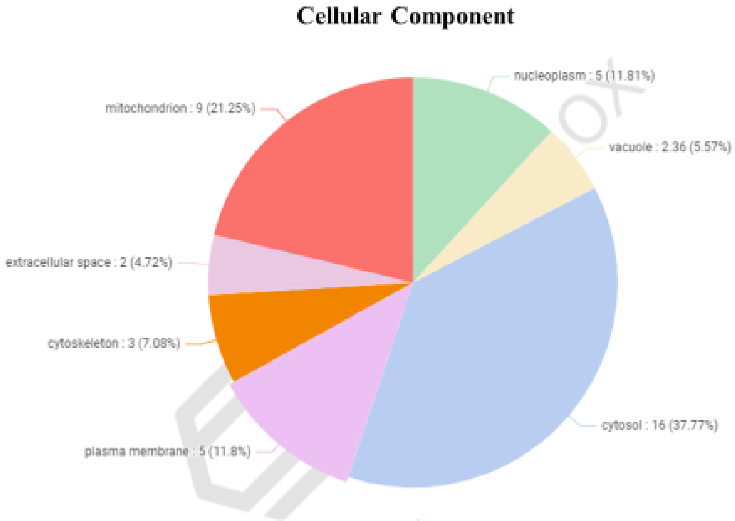
Cellular Component
mitochondrion : 9 (21.25%)
extracellular space : 2 (4.72%)
cytoskeleton : 3 (7.08%)
plasma membrane : 5 (11.8%)
cytosol : 16 (37.77%)
vacuole : 2.36 (5.57%)
nucleoplasm : 5 (11.81%)

(C)

Figure S4. Functional annotation study revealed Volatile Carbon Dioxide's impacts on diverse biological processes (A), molecular functions localization (B), and Cellular Component (C) of distinct proteins in *Homo sapiens*.

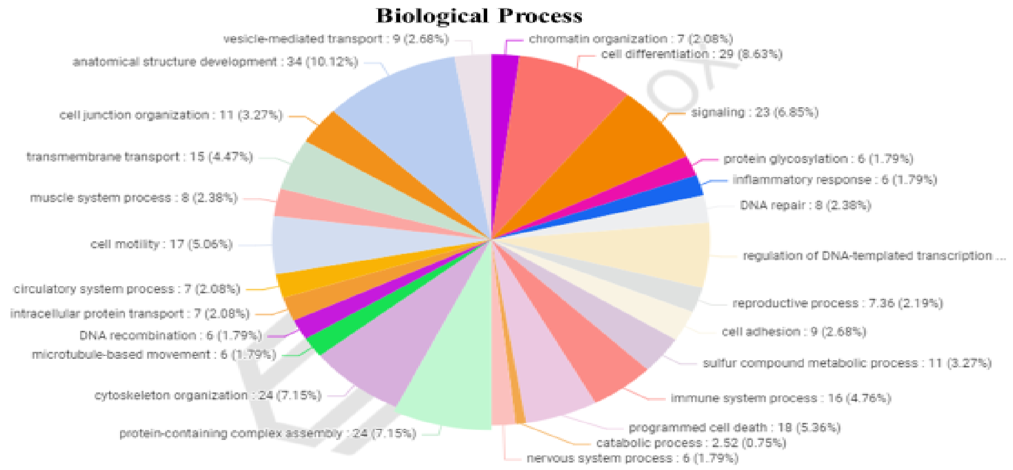
Biological Process
vesicle-mediated transport : 9 (2.68%)
chromatin organization : 7 (2.08%)
cell differentiation : 29 (8.63%)
anatomical structure development : 34 (10.12%)
cell junction organization : 11 (3.27%)
signaling : 23 (6.85%)
transmembrane transport : 15 (4.47%)
protein glycosylation : 6 (1.79%)
inflammatory response : 6 (1.79%)
muscle system process : 8 (2.38%)
DNA repair : 8 (2.38%)
cell motility : 17 (5.06%)
regulation of DNA-templated transcription ...
circulatory system process : 7 (2.08%)
reproductive process : 7.36 (2.19%)
intracellular protein transport : 7 (2.08%)
cell adhesion : 9 (2.68%)
DNA recombination : 6 (1.79%)
microtubule-based movement : 6 (1.79%)
sulfur compound metabolic process : 11 (3.27%)
cytoskeleton organization : 24 (7.15%)
immune system process : 16 (4.76%)
protein-containing complex assembly : 24 (7.15%)
programmed cell death : 18 (5.36%)
catabolic process : 2.52 (0.75%)
nervous system process : 6 (1.79%)

(A)

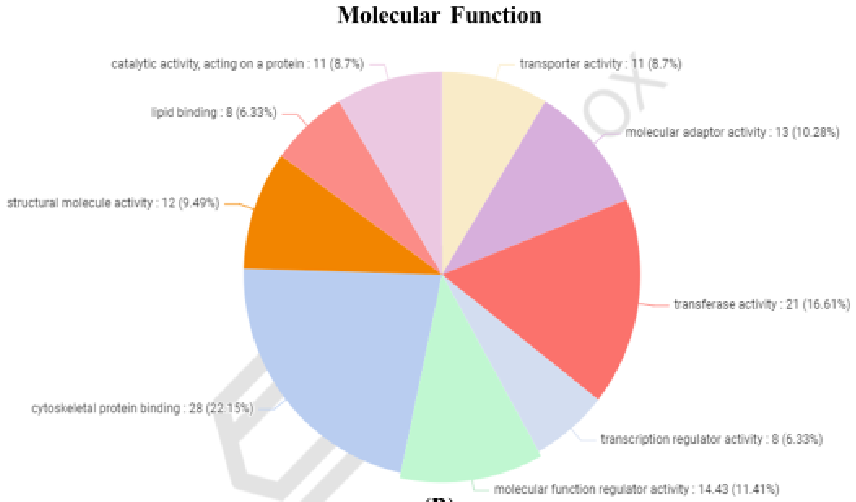
Molecular Function
catalytic activity, acting on a protein : 11 (8.7%)
transporter activity : 11 (8.7%)
lipid binding : 8 (6.33%)
molecular adaptor activity : 13 (10.28%)
structural molecule activity : 12 (9.49%)
transferase activity : 21 (16.61%)
cytoskeletal protein binding : 28 (22.15%)
transcription regulator activity : 8 (6.33%)
molecular function regulator activity : 14.43 (11.41%)

(B)

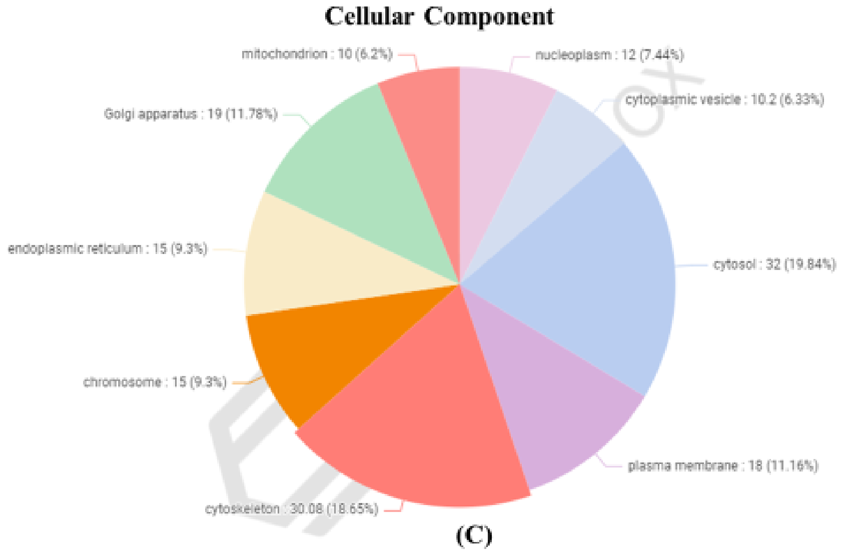

(C)

Figure S5. Functional annotation study revealed SO2's impacts on diverse biological processes (A), cellular component localization (B), and molecular functions (C) of distinct proteins in *Homo sapiens*.

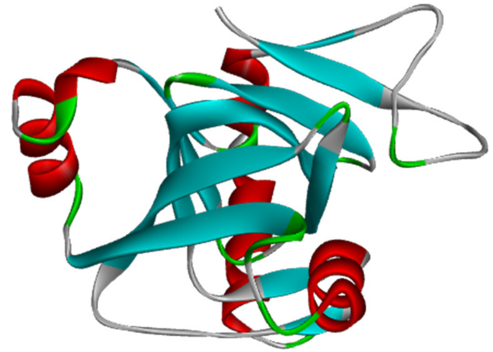

(ADI1)

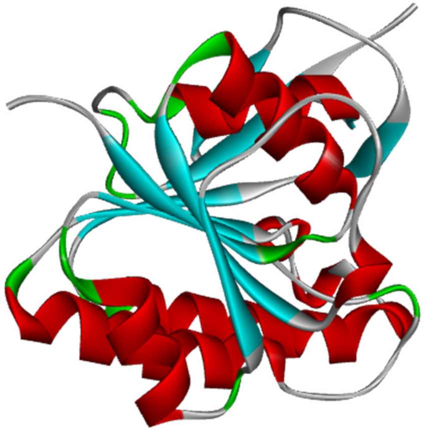

(BLVRB)

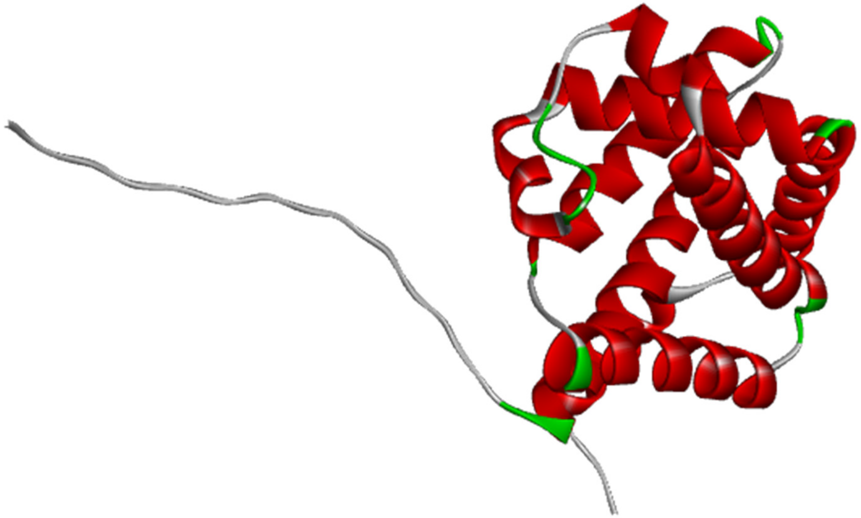

(CYGB)

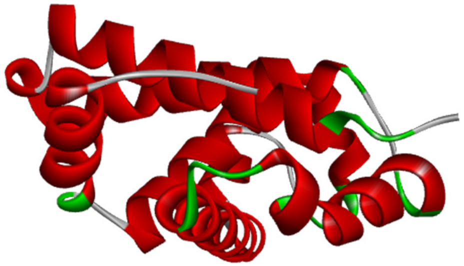

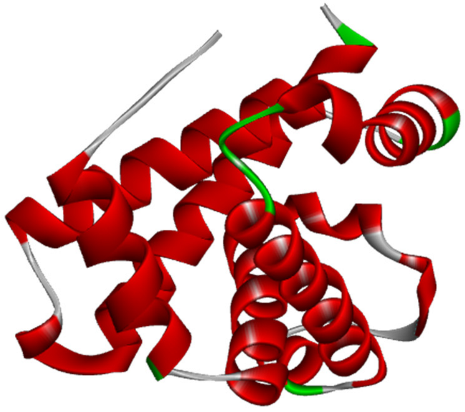

(HBB) **(HBE1)**

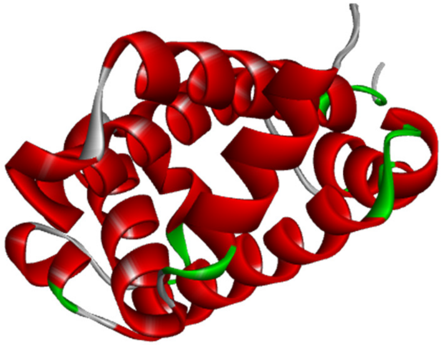

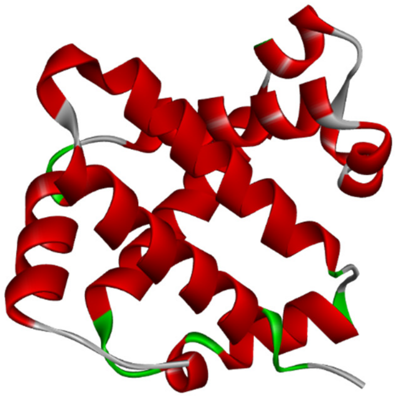

(HBG1) **(HBG2)**

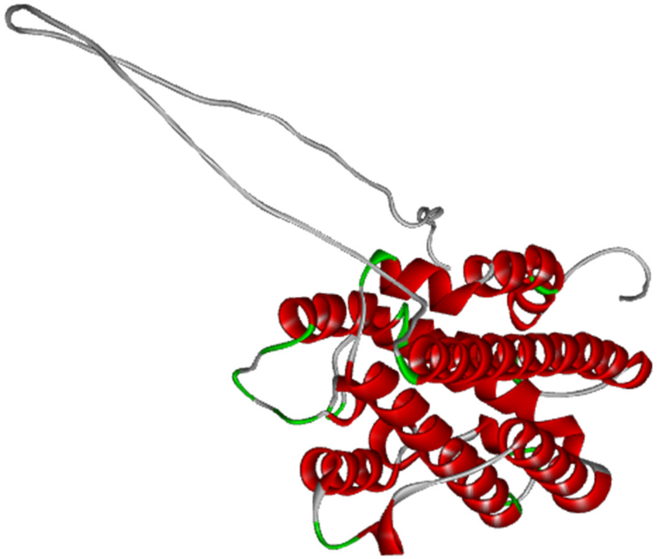

(HMOX1)

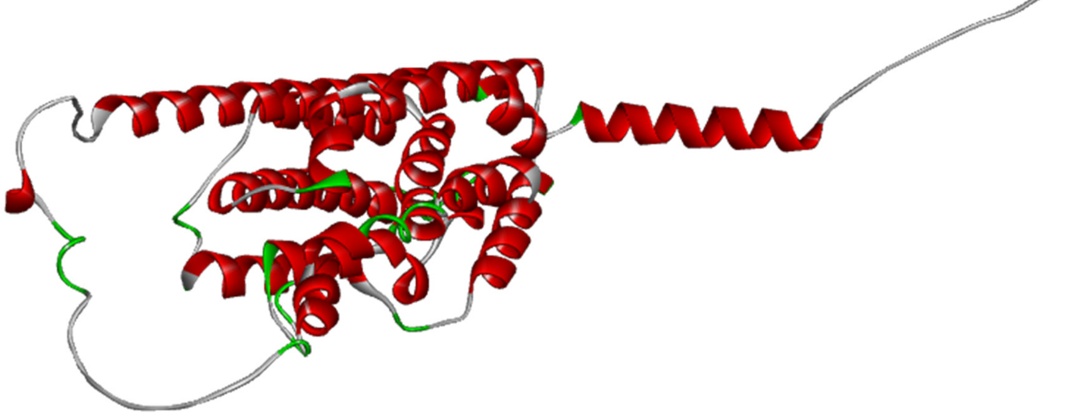

(HMOX2)

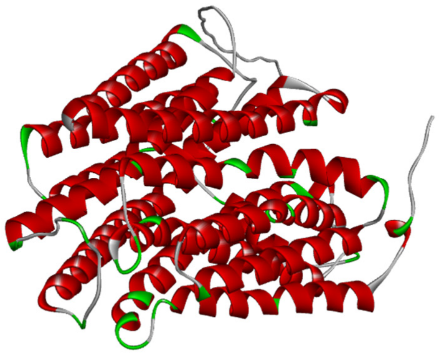

(SLC46A1)

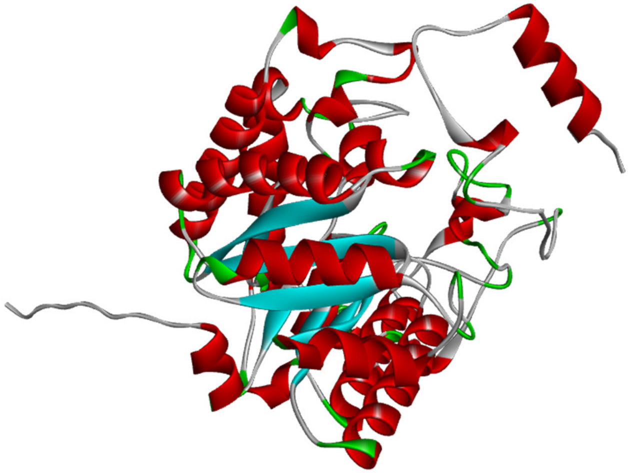

(BHMT)

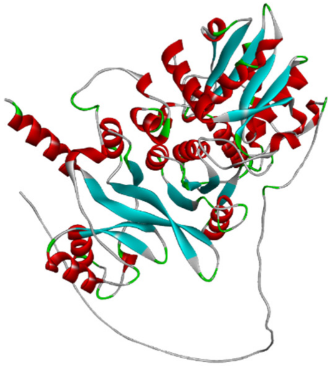

(CBS)

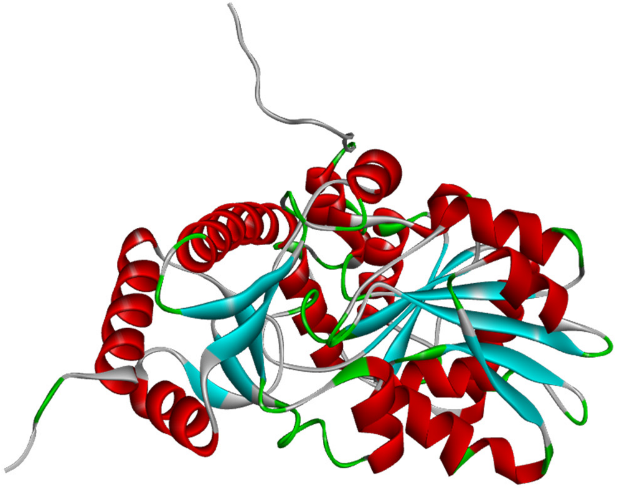

(CTH)

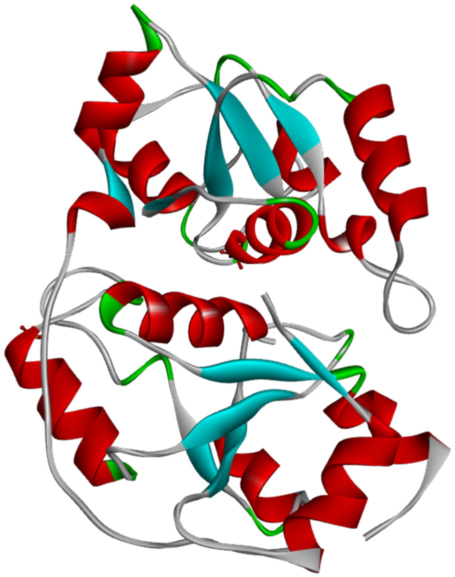

(MPST-3)

Figure S6. 3D structure of proteins that interacted with CO and H_2S identified by STITCH

Table S1. Protein-protein interaction (PPI) of NOx identified through STRING 9.1

SL no.	CAPSAICIN	ENOX1	MBTPS1	TRIM33	SRPK2
1	CALM3	TRIM43	MBTPS2	SMAD2	SRSF1
2	TRPM8	CTIF	ATF6	SMAD3	DDX23
3	CALML3	POP5	SCAP	CDC20	SRSF2
4	CALML5	OR5M11	CREB3L3	ANAPC5	U2AF2
5	TRPA1	C10orf71	CREB3L2	BUB1B	SNRNP70
6	CALML6	PMFBP1	SREBF2	ANAPC1	SRSF6

7	TRPA1	CCDC126	CREB3L1	FZR1	LUC7L
8	CALML6	PMFBP1	CREB3L4	ANAPC7	SRSF5
9	CALML4	CCDC126	SREBF1		U2AF1
10	PRKACA	TMEM161B	CREB3		
11	PRKACB	LHPP			
12	PRKACG	ARHGAP28			

Table S2. Protein-protein interaction (PPI) of Ozone (O_3) identified through STRING 9.1

SL no.	**CFTR**	**GSTM1**	**IL8**	**NOS3**	**SERPINE1**	**SFTPA1**	**SFTPA2**	**TNF**	**TPTE**	**ZNF410**
1	CFTR	CYP1A1	ACKR1	AKT1	AGTR1	AGER	ABCA3	CASP8	AKT1	CDX2
2	GOPC	CYP1A2	CCL2	CALM3	CCL2	DMBT1	ELMOD2	CHUK	AKT2	CNOT1
3	HSP90AA1	CYP1B1	CCL8	CALML3	LRP1	LTF	ERV3	MAP3K7	CTAG1B	CPA2
4	HSPA8	CYP2A6	CCR1	CALML4	PLAT	NKX2	ERVFRD	NFKB1	MAGEA3	CXorf38
5	PRKACA	CYP2C9	CCR2	CALML5	PLAU	SCGB1A1	LTF	RIPK1	MTMR2	CYP24A1
6	PRKACB	CYP2E1	CXCL8	CALML6	PLAUR	SCGB3A2	SCGB1A1	TAB2	MTMR7	CYP2R1
7	PRKACG	CYP3A4	CXCR1	CAV1	PLG	SFTPA1	SFTPA1	TNF	NEK3	ST8SIA4
8	RNF5	EPHX1	CXCR2	HSP90AA1	PROC	SFTPA2	SFTPA2	TNFRSF1A	PTEN	TFCP2
9	SLC9A3R1	GSTM1	CXCR3	HSP90AB1	SERPINB2	SFTPB	SFTPB	TNFRSF1B	SPRTN- DNA	ZNF410
10	SLC9A3R2	GSTM2	IL4	NOS3	SERPINE1	SFTPC	SFTPC	TRADD	TPTE	
11	STUB1- E3	SPP1		NOSIP	VTN	SFTPD	SFTPD	TRAF2	TYR	

Table S3. Protein-protein interaction (PPI) of Volatile Carbon Dioxide identified through STRING 9.1

SL no.	**CA$_1$**	**CA$_2$**	**OGDH**	**PAICS**	**ALDH1L1**	**UROD**	**HBB**	**PCK1**	**PCK2**
1	CYP24A1	SLC9A1	DLD	PPAT	GART	PPOX	HBA2	PC	PC
2	HBD	SLC4A1	DLST	GART	MTHFD1	CPOX	HBA1	PKLR	MDH2
3	AHSP	SLC4A4	SUCLG1	ADSL	SHMT1	UROS	HBZ	ENO1	ENO1
4	CA2	TCIRG1	IDH3A	ATIC	SHMT2	HMBS	AHSP	ACLY	ENO3
5	EPB42	ATP6V1B1	IDH2	CHD7	MTHFD2	ALAD	HBQ1	ENO3	PKLR
6	SLC4A1	CA1	IDH1	MTHFD1	MTHFD2L	FECH	HPR	MDH2	CS

7	ALAS2	CBS	DLAT	ADSS2	ALDH1L2	HFE	HBM	ENO4	ENO2
8	GYPB	CBSL	IDH3B	SHMT1	DHFR	ALAS1	HP	ENO2	ACLY
9	STAT4	CLCN7	IDH3G	GMPS	AMT	MOCS1	HBD	PKM	PKM
10	SELENBP1	CYP24A1	PDHX	SHMT2		ALAS2	BCL11A		

Table S4. Protein-protein interaction (PPI) of Sulphur Dioxide (SO_2) identified through STRING 9.1

SL no.	**ACTA1**	**ACTB**	**ACTBL2**	**ACTG1**	**GSN**	**MAPK8IP1**	**MAPK8IP2**	**SLC35B2**	**SLC35B3**	**UST**
1	ACTA1	ACTB	ACTB	ACTB	ACTA1	APP	FGF12	B3GAT3	B3GAT3	DSE
2	ACTR2	ACTG1	ACTBL2	ACTG1	CTTN	JUN	KLC2	B4GALT7	SLC35A1	EXT1
3	CFL1	ACTL6A	ACTL6A	CFL1	GSN	KLC1	MAP2K3	EXT2	SLC35A4	EXT2
4	CFL2	ARID1A	ACTL6B	CFL2	HCLS1	KLC2	MAP2K7	EXTL3	SLC35A5	GLCE-D
5	GSN	CFL1	DMAP1	MYH14	PFN1	MAP2K7	MAP3K10	RPL15	SLC35B2	HS3ST1
6	LCP1	CFL2	GSN	MYH9	PFN3	MAP3K11	MAP3K11	SLC35B2	SLC35B3	HS3ST3B1
7	PFN1	PFN1	PFDN1	PFN1	PFN4	MAP3K13	MAPK8	SLC35D1	SLC35C1	HS3ST4
8	TNNC2	PFN3	PFDN2	PFN3	TLN1	MAPK8	MAPK8IP1	SLC35D3	SLC35C2	NDST1
9	TPM1	PFN4	RUVBL1	PFN4	TLN2	MAPK8IP1	MAPK8IP2	SULT4A1	SLC35D1	NDST2
10	TPM3	SMARCA4	RUVBL2	VCL	TWF2	MAPK8IP2	MAPK8IP3	TPST2	SLC35D2	NDST3
11	VASP	SMARCE1	TWF2	WASL	WASL	MAPK9	MAPK9		SLC35D3	

Table S5. Degree centrality, closeness centrality, and betweenness centrality of CO interacted proteins identified by STRING

Protein Name	Degree	Betweenness	Closeness	Protein Name	Degree	Betweenness	Closeness	Protein Name	Degree	Betweenness	Closeness
ACP5	4	0.014	0.433	FOLR2	3	0.021	0.374	HCCS	6	0.001	0.436
CRP	10	0.006	0.473	FOLR3	3	0.021	0.374	CYBRD1	7	0.001	0.430
ALB	24	0.280	0.565	HPR	9	0.005	0.442	SLC11A2	9	0.001	0.439
BLVRB	17	0.106	0.513	NQO1	10	0.018	0.496	HEPH	7	0.000	0.433
RFK	3	0.001	0.386	KEAP1	10	0.003	0.477	FXN	13	0.030	0.484
ADGB	2	0.000	0.377	FOLR1	2	0.021	0.372	FLVCR1	5	0.000	0.418
NGB	9	0.021	0.477	NFE2L2	10	0.005	0.496	GART	4	0.033	0.280
FECH	26	0.144	0.565	TAT	2	0.033	0.367	RNASEH1	1	0.000	0.219
AHSP	15	0.006	0.469	GPX3	16	0.021	0.530	HCCS	6	0.001	0.436
HBQ1	15	0.006	0.477	GPT	11	0.003	0.477	CYBRD1	7	0.001	0.430
HBA2	16	0.013	0.480	F2	7	0.001	0.436	SLC11A2	9	0.001	0.439
HBZ	18	0.019	0.488	HP	21	0.048	0.540				
KLF1	17	0.026	0.517	APIP	2	0.000	1.000				
HBE1	19	0.028	0.530	MRI1	2	0.000	1.000				
BCL11A	13	0.002	0.427	ENOPH1	2	0.000	1.000				
NFE2	16	0.025	0.492	HBS1L	8	0.001	0.396				
GATA1	17	0.026	0.517	MIPEP	8	0.019	0.452				
HBM	13	0.001	0.424	HARS2	3	0.000	0.381				
HBG2	16	0.005	0.452	POR	8	0.007	0.433				
HBG1	17	0.020	0.488	HMOX2	17	0.029	0.513				
HBA1	15	0.009	0.469	MT-ND6	9	0.000	0.367				
HBD	15	0.004	0.477	MT-ND5	9	0.000	0.367				
HMOX1	25	0.123	0.598	MT-ND1	9	0.000	0.367				
EPO	16	0.029	0.526	NDUFS8	9	0.000	0.367				
CP	15	0.029	0.517	NDUFV1	11	0.007	0.412				
BLVRA	10	0.024	0.517	CYC1	13	0.018	0.430				
MMP14	1	0.000	0.363	UQCRFS1	14	0.017	0.436				

Table S6. Degree centrality, closeness centrality, and betweenness centrality of H_2S interacted proteins identified by STRING

Protein Name	Degree	Betweenness	Closeness
GOT1L1	8	1.044	17
GOT2	12	6.227	19
SUOX	12	6.710	19
NFS1	8	0.667	17
GOT1	13	5.718	19.5
ETHE1	9	1.716	17.5
MOCS3	6	0.000	16
MPST	15	15.263	20.5
TST	15	15.263	20.5
MTR	19	9.203	22.5
BHMT	19	9.203	22.5
CBS	24	44.935	25
CTH	25	43.571	25.5
BHMT2	17	3.666	21.5
AHCYL1	13	0.000	19.5
AHCYL2	15	0.308	20.5
CBSL	24	44.935	25
MAT1A	20	15.390	23
SHMT2	19	12.352	22.5
MAT2A	19	12.902	22.5
MTHFR	17	3.666	21.5
SHMT1	18	6.198	22
MTHFD1	17	7.318	21.5
MTRR	14	0.000	20
DMGDH	15	0.143	20.5
CDO1	14	8.499	20
AHCY	17	3.105	21.5
